# Supplementary material for: Inflammatory factors and risk of lung adenocarcinoma: a Mendelian randomization study mediated by blood metabolites
Source: Front Endocrinol (Lausanne). 2024 Aug 27;15:1446863. doi: 10.3389/fendo.2024.1446863 (PMC11384989; doi:10.3389/fendo.2024.1446863)
Supplement: Supplementary file 1 [file DataSheet1.zip › supplementary files/S2/GCST90200582/sensitivity-analysis.pdf]

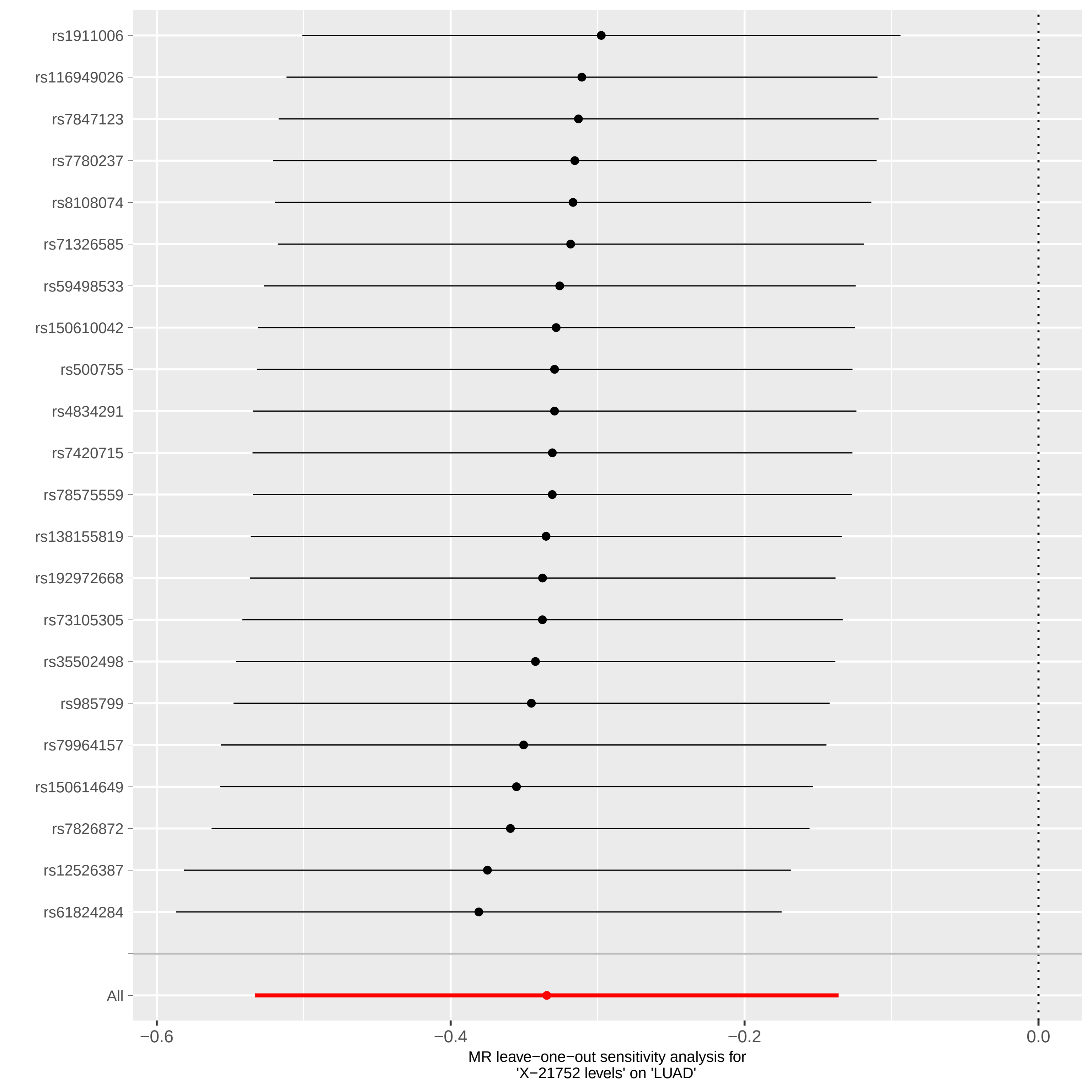
rs1911006
rs116949026
rs7847123
rs7780237
rs8108074
rs71326585
rs59498533
rs150610042
rs500755
rs4834291
rs7420715
rs78575559
rs138155819
rs192972668
rs73105305
rs35502498
rs985799
rs79964157
rs150614649
rs7826872
rs12526387
rs61824284
All
−0.6
−0.4
−0.2
0.0
MR leave−one−out sensitivity analysis for
'X−21752 levels' on 'LUAD'
